# Mechanical Safety of Embedded Electronics for In-body Wearables: A Smart Mouthguard Study

**DOI:** 10.1007/s10439-019-02267-4

**Published:** 2019-04-25

**Authors:** Helen Bridgman, Man Ting Kwong, Jeroen H. M. Bergmann

**Affiliations:** Natural Interactions Lab, Oxford Institute of Biomedical Engineering, Department of Engineering Science, University of Oxford, Old Road Campus Research Building, Oxford, OX3 7DQ UK

**Keywords:** Mechanical assessment, Impact, Embedded electronics, Thermoformed, FEM

## Abstract

The growing popularity of contact sports drives the requirement for better design of protective equipment, such as mouthguards. Smart mouthguards with embedded electronics provide a multitude of new ways to provide increased safety and protection to users. Characterisation of how electronic components embedded in typical mouthguard material, ethylene vinyl acetate (EVA), behave under typical sports impacts is crucial for future designs. A novel pendulum impact rig using a hockey ball disc impactor was developed to investigate impact forces and component failure. Two sets of dental models (aluminium and plastic padding chemical metal) were used to manufacture post-thermoformed mouthguards. Seven embedding conditions with varying thickness of EVA (1.5 and 3 mm) and locations of electrical components were tested. Component failures were observed in four out of seven test conditions, and the experimental failure forces at which the electrical component had a 50% chance of failure were reported for those cases. The experimental results showed that an EVA thickness of 3 mm surrounding the electrical component gives the most comprehensive protection even under extreme surface conformity. Computational models on surface conformity of EVA showed that a block of EVA with a minimum thickness of 1.5 mm was better at reducing stress concentration than a shell with an overall thickness of 1.5 mm. This study demonstrated that the thickness of a mouthguard is important when protecting electrical components from extreme dental surface conformity, furthermore the surface geometry should not be overlooked when considering electrical component safety for in-body wearables that are impact prone.

**Electronic supplementary material:**

The online version of this article (10.1007/s10439-019-02267-4) contains supplementary material, which is available to authorized users.

## Introduction

It is estimated that physical inactivity cost the global health-care systems $53.8 billion annually^7^ and the World Health Organisation (WHO) states that approximately two million deaths per year are attributed to physical inactivity. In the UK, the government target is to have adults take part in at least 150 min of moderate activity or 75 min of vigorous activity per week.^25^ Physical activity through sports participation has become an essential part in ensuring healthy living across the globe, with some of the biggest and fastest growing sports being contact-sports. These sports also create significant economic outputs, with the Rugby World Cup 2015 producing £2.3 billion from a single event and American Football being the highest revenue generating professional sports league in the world.

The popularity of contact sports and extreme sports has increased exponentially over the past two decades, despite the increased risk of injuries associated with these sports.^17^ A third of all facial injuries is caused by sporting activities and 50% of these are oral or dental.^9^ Consequently, there is increasing pressure to improve player safety through both rule changes and by promoting broader application of safety equipment. A meta-analysis in 2007 evaluated the effectiveness of mouthguards in reducing dental injuries and found the overall risk of injury reduced by a factor of 1.6–1.9 if a mouthguard was worn.^24^ Mouthguards work by dissipating the force of impact, thus reducing the force which is transferred to the dentition.^24^ They are commonly formed to the upper jaw, as this region is more susceptible to trauma. The wide advocacy of mouthguard use has led to their adoption as mandatory equipment in several sports.^14^

Embedding electronics into the mouthguard allows for unobtrusive, remote monitoring of physiological parameters, as athletes are already accustomed to using this piece of equipment during sports participation. The types of data which could be collected at the point of the oral cavity is vast and can positively affect injury rates. Currently, the primary focus of these “smart” mouthguards have been on measuring impact to enable coaches to identify athletes needing side-line concussion protocol testing.^10^ It is therefore important that the electronic components are performing in a reliable fashion. Instrumented mouthguards have previously been shown to better capture head impact acceleration than an instrumented helmet.^12^ Instrumented mouthguards have been developed to monitor both impacts in an *in vivo* and *in vitro* environment.^3^ Such mouthguards are able to measure the magnitude, direction of impacts typical in contact sports such as American football,^5^,^11^,^32^^–^34 boxing^3^ and soccer.^8^ Furthermore, a study by Kuo *et al*.^16^ showed that it is advantageous to embed sensors in front of the incisor rather than adjacent to the rear molar to reduce the sensor kinematic errors as a result of mandibular forces. However, no work was reported on how impacts to the incisor would affect the functioning of the embedded components. There are currently no studies that measure the amount of impact forces that a typical electrical component can withstand when embedded into mouthguards or similar protective equipment. Furthermore, current impact tests are performed on pre-formed sheets and do not take into account material thinning from thermoforming thus limiting the external validity of these results in practice. A more suitable approach would be to test a post-formed mouth-guard.^8^

A review of twenty-six research studies on impact testing for mouthguards found that in over 75% of the studies, the force applied to the mouthguard originated from either a pendulum or drop-ball.^14^ In over 65% of impact tests a steel object was used as the impactor. Yet, sports-related trauma is rarely caused by steel objects and is instead inflicted by various sized balls, bats, sticks or gloves, which are produced from a diverse range of materials. It has previously been shown that steel impactors produce an unrealistic impact and it is therefore important to test mouthguards with sport specific materials.^29^

Shock absorbing capability can be broadly defined as the reduction in impact energy or force transmitted to the surface beneath the mouthguard. For drop-ball studies, this is typically calculated from the rebound height of the impactor. Alternatively, a force transducer is placed beneath the mouthguard material and a known force is applied to the top of the material or otherwise an accelerometer can be utilised to measure the acceleration of the impactor to find the impact force.^14^ The magnitude of the applied force varies significantly between studies because the parameter of concern is the relative reduction in force transmitted.

Mouthguard material is very ductile and does not yield under common sports impacts. Therefore, mouthguard research often focuses on relative reduction in impact energy rather than a specific failure force. However, it is important to understand the range of forces caused by a typical impact at which embedded electronics could yield.

The aims of this study are (i) experimentally determine a failure force (*F*_fail_) at which a typical electrical component used in smart mouthguard designs would structurally fail. Failure force was selected (as oppose to strain base), as forces can be more readily evaluated in a on field scenario in terms of video replays, and therefore can be more readily translated for the use of practical management of component safety. (ii) Investigate the thickness of mouthguard material necessary to protect the electrical components and (iii), explore the effect surface conformity has on the electrical component’s failing.

## Materials and Methods

### Experimental Setup

**Figure 1:**
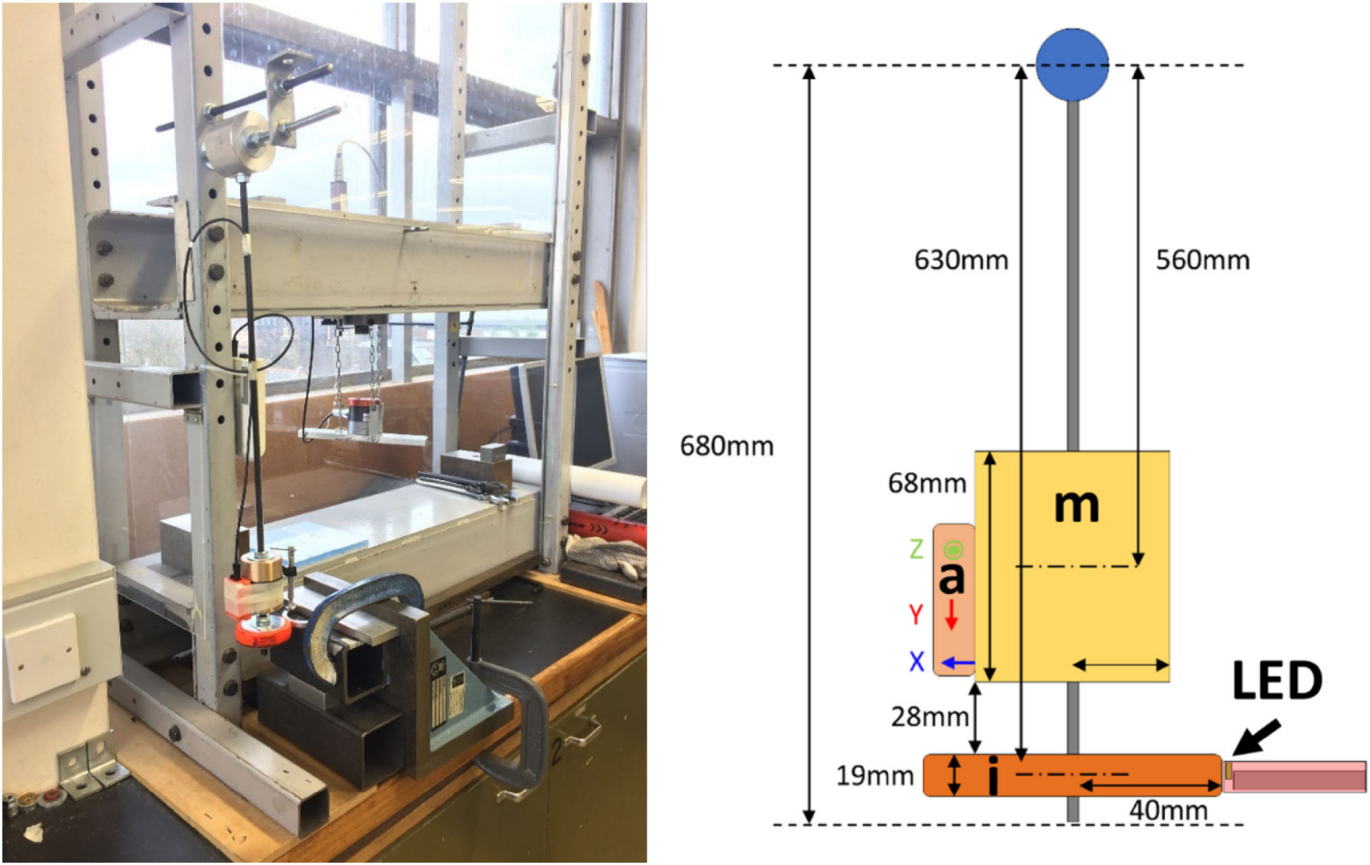
Image (left) and a schematic (right) of the pendulum test rig. A mass (m) and an accelerometer (a) are mounted directly above the impactor (i) to reproduce ball impact in hockey games. The electronic component, a light-emitting diode (LED) is mounted directly on the dental model, on which the mouthguard is mounted.

A pendulum impact rig was used to reconstruct the impact load of a ball-mouthguard collision. Fully thermoformed mouthguards with embedded electronic components were impacted by a hockey ball disc impactor with the mouthguard mounted on a dental cast. A schematic of the apparatus configuration is depicted in Fig. 1.

Impact testing was performed by releasing the pendulum from a range of angles (0°–86°) and allowing it to collide with the fixed mouthguard and teeth (fixed by clamping the top and the base). The supplementary information provides more information on the impactor and impact acceleration. The pendulum was released manually from a stationary position and care was taken to prevent the transfer of any additional kinetic energy during release. An inertial measurement unit (MTx, Xsens Technologies B.V., Enschede, Netherlands) was used to record the angle articulated by the pendulum, the angular velocity on impact, and the impulse time. After each impact, LED was inspected. If the LED was fragmented or cracked, it was categorised as ‘Failed’, otherwise it was categorised as ‘Intact’.

An explorative approach was used to determine the exact release angle at which the electrical component would fail, thus ensuring that there were enough data points for which the component transitioned from ‘Intact’ to ‘Failed’. Twenty-five impacts were performed on various LED embedding arrangements.

### Dental Models

**Figure 2:**
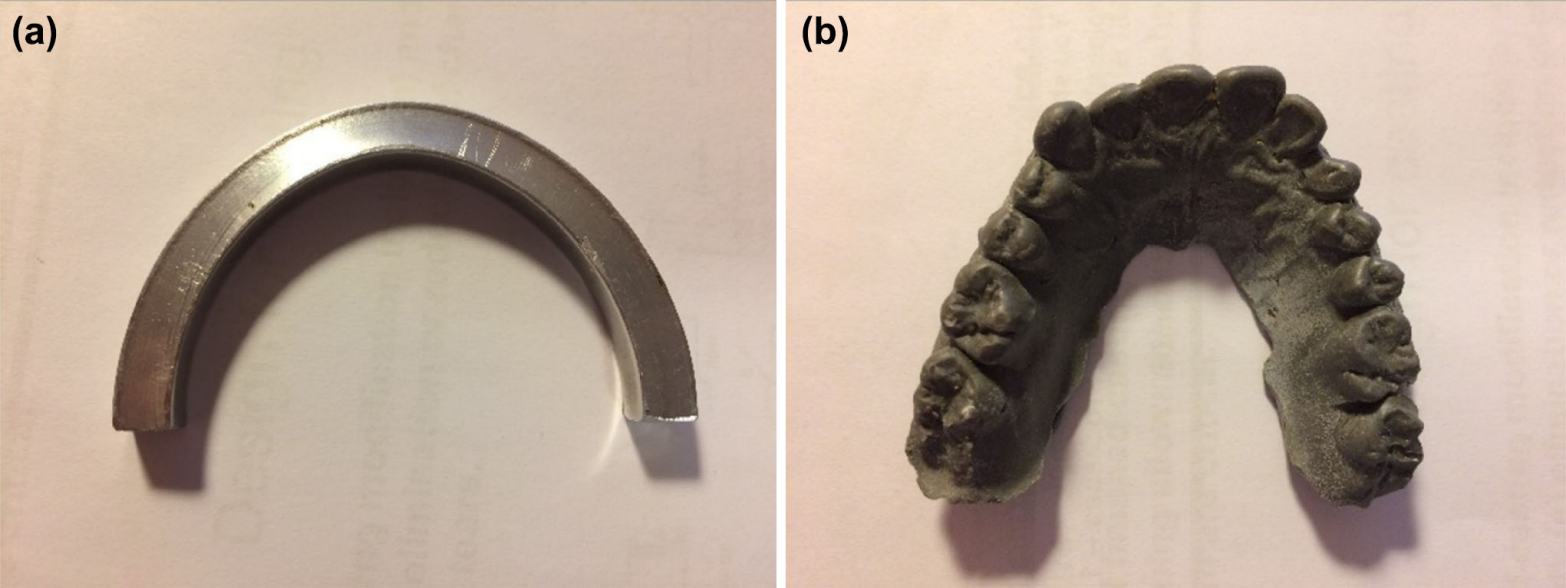
Top-view of the two dental model. Model A (left) is a simplified dental model constructed in aluminium. Model B (right) shows a dental model constructed through a dental cast using Plastic Padding Chemical Metal.

Two sets of dental models were used to represent teeth enclosed by the mouthguard during a ball-mouthguard collision (Fig. 2). Model A consisted of a semi-circular annular ring of aluminium, which was based on a reported average total arch length of 42.6 mm, average inter-second molar distance of 56.9 mm^1^ and average tooth labial thickness of 6 mm.^4^ Model B consisted of a cast of the maxilla (the upper jaw) using Plastic Padding Chemical Metal (Loctite, Düsseldorf, Germany). The chemical metal has an ultimate failure strength of 90 MPa,^18^ which is within the range of compressive strength for enamel and dentin and therefore makes it a suitable material for dental models.^38^

### Mouthguards

Ethylene vinyl acetate (EVA) is the most commonly used material for mouthguard fabrication due to its formability, ease of manipulation, and shock absorbing ability.^14^ Transparent pro-form soft EVA vacuum forming disks were selected to ease identification of failed components under the EVA. The disks had a diameter of 120 mm and a thickness of 1.5 mm. A vacuum-forming process (Vacfomat-U, Dreve Dentamid GmbH, Germany) was applied for the fabrication of the mouthguards, combined with a heating temperature within the range of 80–120°C.^35^ The disks were heated for 1.5 min at which they became visibly more fluid and viscous. They were then pressed onto the models and the vacuum was applied. A 10-min interval between the forming of each mouthguard was taken to allow for the cooling of the EVA to achieve consistent moulding.^28^ A selection of mouthguard thicknesses ranging from 1.5 up to 6 mm (pre-formed) were tested (see supplementary material for more information).

### Embedded Electronics

Mouthguards may be fitted with a broad range of different electronic components with varying functions and physical specifications. The EVA material acts as a physical barrier between the electronics and the environment. Mechanical stress induced failure of integrated electrical components is particularly important in the context of ball-mouthguard collisions. Structural failure of the electronic components often signifies function failure. In most cases the time at which impact occurs might be the most relevant period of measurement. The reliability of the electronic components under impact is therefore an important design criterion.

Through-hole LEDs are fabricated by potting a small electronic assembly into a solid compound. An LED was selected as a representative electrical component for the impact tests due to the component’s relative homogenous material properties, simple geometry, wide application base, and ease to detect failure mode. A rectangular shaped LED produced by Kingbright with an epoxy resin potting compound (dimensions: 7 mm × 5 mm × 2 mm) was selected.^13^ The rectangular shape of the component ensured repeatability in terms of orientation during testing and the rectangular form is an often occurring shape for electrical components. Due to the translucent nature of LED lenses, crack propagation could be easily observed, and a simple circuit was used to gauge whether the component’s performance/function was compromised by the impact.

### Experimental LED Embedding Conditions

Seven different embedding conditions were tested, where the thickness of EVA and the vertical offset of the LED position relative to the dental casts were varied (here vertical offset was used as a proxy for all surface conformity). Table 1 provides an overview of the different conditions.

**Table 1:** Summary of embedding arrangement parameters for the seven conditions.

Condition	1	2	3	4	5	6	7
Dental model	A	A	B	A	A	B	B
EVA thickness (mm)	0	0	0	1.5	3	1.5	3
LED position	No offset	3.5 mm offset	Central to incisor	3.5 mm offset	3.5 mm offset	Central to incisor	Central to incisor

**Figure 3:**
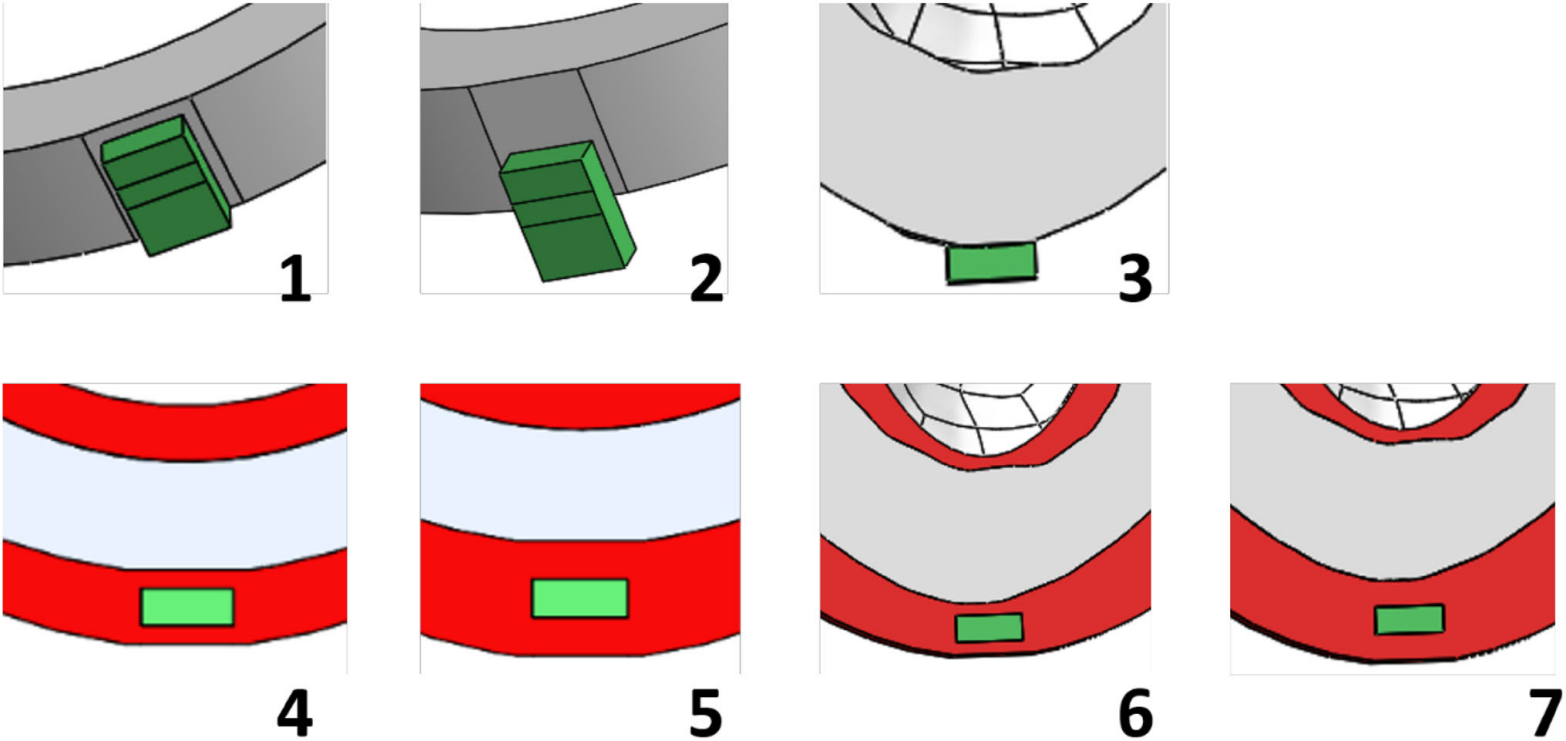
Schematic of the seven LED embedding conditions. The LED is coloured green, the EVA material is red, and the dental models are grey.

In terms of the vertical offset of the LED relative to the dental cast, 0 and 3.5 mm overhangs were tested for model A (see Fig. 3, conditions 1 and 2). LEDs were placed central to the incisor with no offset on model B (condition 3), as this is the most common location for sports-related dental trauma.^2^ In terms of EVA thickness, thicknesses of 1.5 and 3 mm were positioned on both sides of the LED for both models A and B (conditions 4–7). These thicknesses were chosen as they are common thicknesses used in mouthguard fabrication.

### Experimental Failure Force

Logistic regression curves were produced for all conditions in which more than one failure was recorded to provide an impact force at which the probability of LED survival was 50%, this force was termed hereafter “experimental failure force” (*F*_fail_). The probability (*p*) was determined by,

1 $$\begin{array}{*{20}c} {\ln \frac{p}{1 - p} = \beta_{0} + \beta_{1} F_{\hbox{max} } } \\ \end{array}$$

which was reorganised to,

2 $$\begin{array}{*{20}c} {p = \frac{1}{{1 + e^{{ - (\beta_{0} + \beta_{1} F_{\hbox{max} } )}} }}} \\ \end{array}$$

*p* = 0.5 was used to minimize the misclassification rate and hence yielding the decision boundary between “Failure” and “Intact”. Newton’s method is used to iteratively determine coefficients *β*_0_ and *β*_1_ from each condition’s twenty-five impact data points. *F*_fail_ can be be expressed as,

3 $$\begin{array}{*{20}c} {F_{\text{fail}} = - \frac{{\beta_{0} }}{{\beta_{1} }} } \\ \end{array}$$

### Computational Model

To complement the experimental investigations, and to gain insight into embedded designs explicit dynamics finite element (FE) simulations using commercial FE software ABAQUS (Dassault Systèmes, France) were used to investigate (i) the stress concentrations in condition 2 and (ii) the effect of mouthguard conformity by comparing the stress concentration between EVA with overall and minimum thickness of 1.5 mm. Condition 2 was selected to provide early insights, as there was a positional offset of the electrical component which can represent all surface conformities.

**Figure 4:**
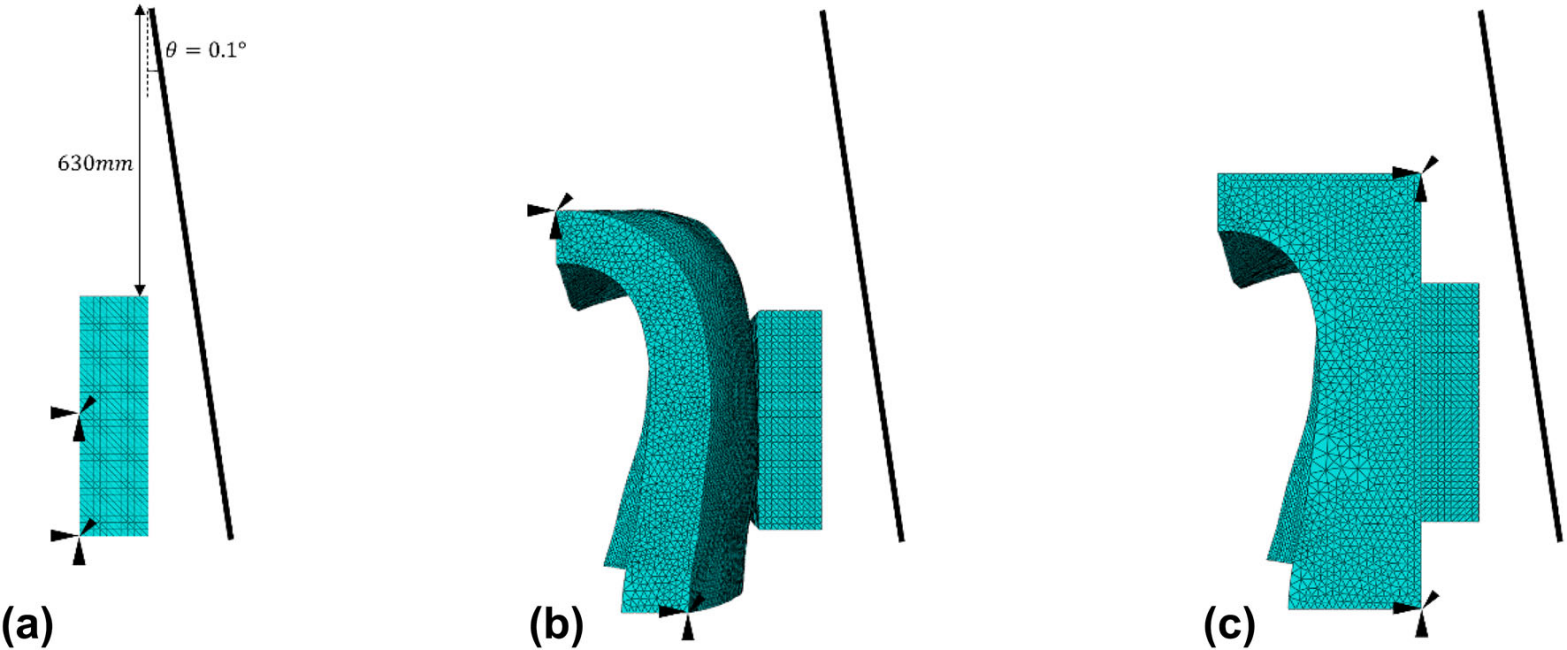
Mesh and boundary conditions for three simulations (a) condition 2—LED mounted on a vertical offset of 3.5 mm to dental model A, (b) an LED mounted on an EVA shell with an overall thickness of 1.5 mm, and (c) an LED mounted on an EVA block with a minimum thickness of 1.5 mm. The impactor was simulated by a rigid plate rotated 0.1° about a reference point 630 mm directly above the LED.

As shown in Fig. 4a, the impactor was simulated as a rigid plate with a mass of 1.811 kg. In order to simplify the simulations, the acceleration phase before the impactor reached the EVA/LED is not explicitly modelled. The impact is characterised by the velocity at the time of the beginning of the impact and the rotational inertia of the impactor. The impact was simulated with an initial angular velocity of 2 rad/s. This angular velocity was the mean angular velocity at impact across the twenty-five tests conducted for condition 2 with a range of 1.06–2.62 rads/s. All the simulated models were subjected to the same impact. Table 2 summarises the simulation study and their purposes.

**Table 2:** Summary of simulation studies.

Simulation description	EVA thickness	Qualitative information
LED on dental model A (condition 2)	0 mm	Component stress concentrations under worst-case impact
LED on uniform EVA layer on dental model B	1.5 mm	Component stress concentrations when mounted on surface with realistic dental conformity
LED on EVA layer on dental model B	Minimum 1.5 mm	Component stress concentrations when mounted on surface with levelled conformity

For studying the stress concentrations in condition 2, the LED was modelled as an isotropic linear elastic cuboid (2 mm × 5 mm × 7 mm) of epoxy resin (Young’s modulus: 2415 MPa,^27^ material density: 1300 kg/m^3^^27^ and Poisson’s ratio: 0.35^27^). The lower back of the LED was assumed to be fully bonded to model A and was simulated as being totally fixed in displacements. The front of the LED was positioned facing the impactor.

For studying the effect of conformity, two EVA geometries (an EVA shell and block) were created using commercial software (Solidworks, Dassault Systèmes, France). The EVA shell was created from a 3D scan of model B, a section approximating the location of experimental placement of the LED (central to incisor) was used to generate a virtual mould. The virtual mould of model B was then extruded by 1.5 mm to create the geometry for the EVA shell, and the resulting meshed geometry is shown in Fig. 4b. Using the same virtual mould, an EVA block was created by projecting a face at least 1.5 mm parallel to the vertical plane of the virtual mould, resulting in geometry shown in Fig. 4c. Since the focus of this study was on the surface conformity of the EVA structures, the EVA was nominally assigned the following isotropic material properties: Young’s modulus of 25 MPa,^20^ material density of 950 kg/m^3^^20^ and Poisson’s ratio of 0.4.^6^ The geometries of the EVA structures were considered by fixing the displacements of the surfaces on the front face of the EVA structure (surfaces facing the LED), and essentially behaved as rigid bodies. The LEDs were located centrally, 2 mm from the base of each mould. The LEDs were assumed to be adhered to the EVA and a bonded contact was prescribed to any interfaces between the LED and EVA structure. The posterior of the EVA structures was fixed in displacements. A global element size of 0.2 mm was used, and the LED was discretised with 55340 quadratic tetrahedral elements in all three simulations.

## Results

**Figure 5:**
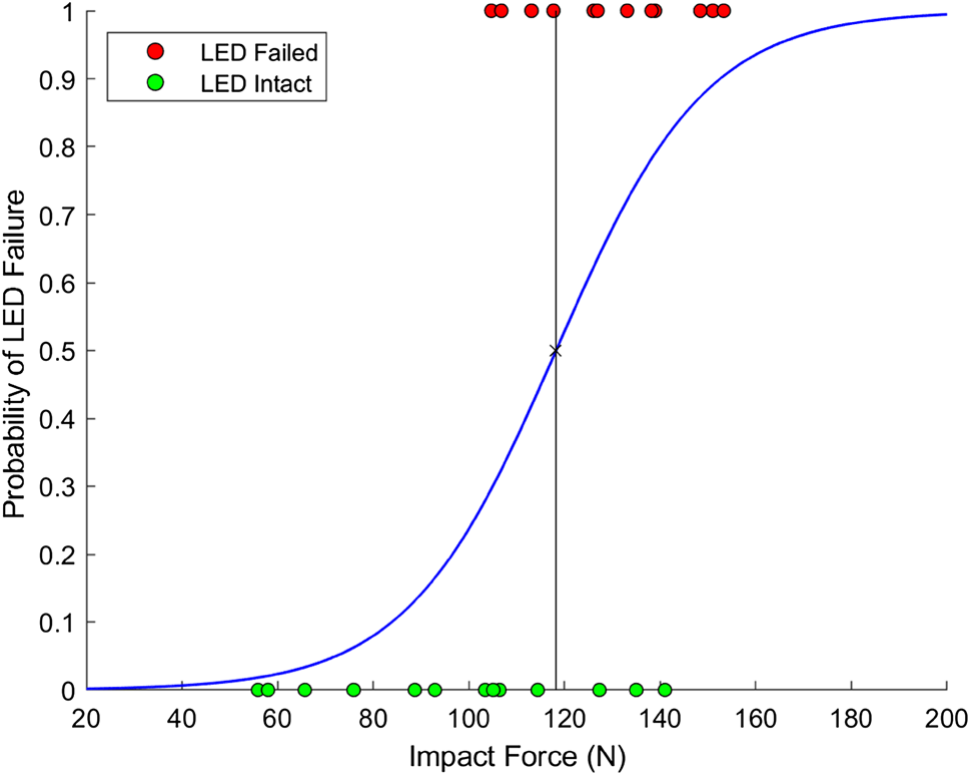
Logistic regression resulting from twenty-five impacts for condition 2.

Experimentally determined values for *F*_fail_ were evaluated for all conditions where structural failure occurred using logistic regression. Figure 5 presents the logistic regression plot for condition 2, approximating the *F*_fail_ at 117.2 N, a summary of the *F*_fail_, *β*_0_ and *β*_1_ for all the conditions are summarised in Table 3.

**Table 3:** Summary of *F*_fail_ and logistic regression coefficients.

Condition	1	2	3	4	5	6	7
*F*_fail_ (*N*)	Intact	117.2	195.7	272.2	Intact	195.7	Intact
*β*_0_	N/A	− 7.567	− 12.857	− 53.049	N/A	− 12.857	N/A
*β*_1_	N/A	0.064	0.066	0.195	N/A	0.066	N/A

If the component did not fail, the result for that condition was labelled as “Intact”

### Protection Study

Impact studies with (i) no mouthguard, (ii) thin (1.5 mm) and (iii) thick (3 mm) EVA protection on both sides of the LED component were performed on dental model A to investigate the amount of EVA protection required to structurally protect electrical components of a smart mouthguard. The condition of no mouthguard protection with no offset placement of the LED (condition 1) was tested and resulted in no fracture in the LED under all experimental impacts. This condition was therefore used as the baseline for minimum protection required to keep the LED component intact. Since increasing the thickness of EVA did not improve protection of the LED for the conditions with no offset placement, conditions with a 3.5 mm offset placement of LED (conditions 2, 4 and 5) were explored.

The current apparatus configuration (impact force capacity of 300 N) was unable to induce LED failure in conditions 1, 5 and 7. LEDs placed at a vertical offset of 3.5 mm on model A with no EVA protection (condition 2) and EVA protection (condition 4) were observed to have a *F*_fail_ of 117.2 N and 272.2 N respectively. LEDs did not fail under experimental impact when protected with thick EVA (condition 5).

LEDs were placed central to the incisor with no offset on model B, as this is the most common location for sports-related dental trauma.^2^ LEDs with no and thin EVA protection (conditions 3 and 6) failed at the same *F*_fail_. LEDs with thick protection mounted on model B remained “Intact” under impact, as similar to those mounted on model A.

**Figure 6:**
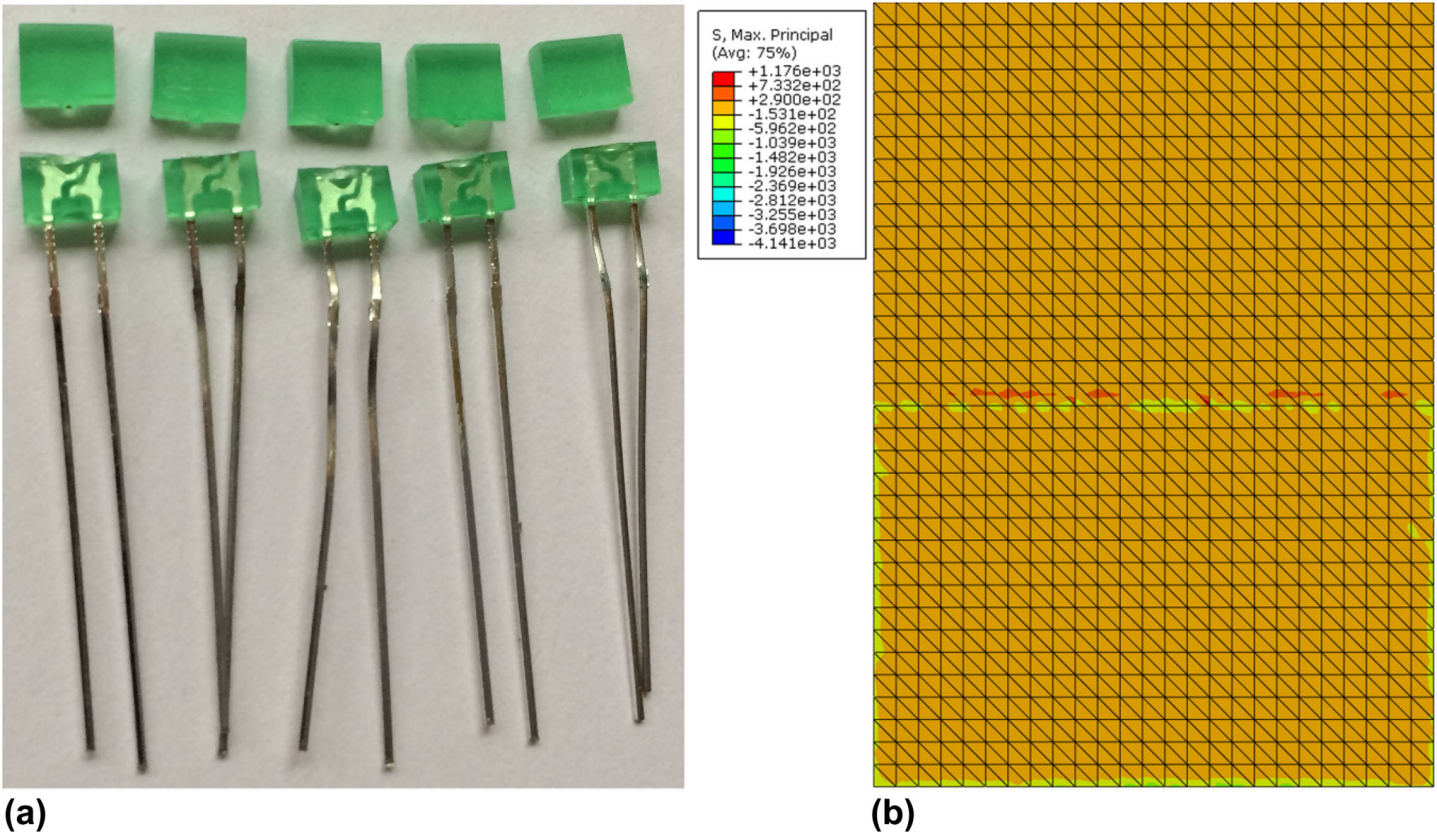
(a) LEDs in condition 2 experience total fracture after experimental impacts. (b) Principal stress distribution of the back of the LED for condition 2 in the original configuration. High stress concentration can be observed at the interface of the LED and the dental model. A maximum principal stress of 1176 MPa was observed at 0.79 ms.

FE models were used to verify the stress concentration of an LED under impact and they showed to be closely aligned with the pattern of fracture observed experimentally. Experimental fracture patterns of LED for condition 2 are presented in Fig. 6a, along with the computed stress concentration (Fig. 6b). The maximum principal stress (1176 MPa) was observed on the back of the LED, near the boundary of where it was fixed to model A (simulated by a fix in displacements).

### Dental Surface Conformity Study

The effect of non-uniform surface conformity was tested by placing the LED component at a 3.5 mm vertical offset to the dental cast. This offset placement aims to capture the effect of worst-case component placement within the smart mouthguard, since high stress concentrations can be expected for such cantilevered region. LEDs placed centrally on dental model A (condition 1) represents the most even surface possible, and as previously mentioned, remained “intact” under impact tests. LEDs mounted on surfaces with the most extreme conformity (condition 2) failed under the lowest *F*_fail_ as expected.

Model B provides a more realistic representation of surface conformity. LEDs under this configuration (condition 3) have shown to withstand a higher experimental *F*_fail_, and they also exhibited a more complex pattern of fracture compare to the most extreme case (condition 2). Furthermore, thin EVA protection on model B (condition 6) did not improve the outcome for the LED component, while thin EVA protection previously showed to improve *F*_fail_ for LEDs mounted on surfaces with the most extreme conformity (condition 4) on model A.

FE models were used here to test the effect surface conformity has on the stress concentration of the LED under impact. Two conditions were tested: (i) LED mounted on a shell of EVA with an overall thickness of 1.5 mm moulded from model B, and (ii) LED mounted on a block of EVA with a levelled surface moulded from model B.

**Figure 7:**
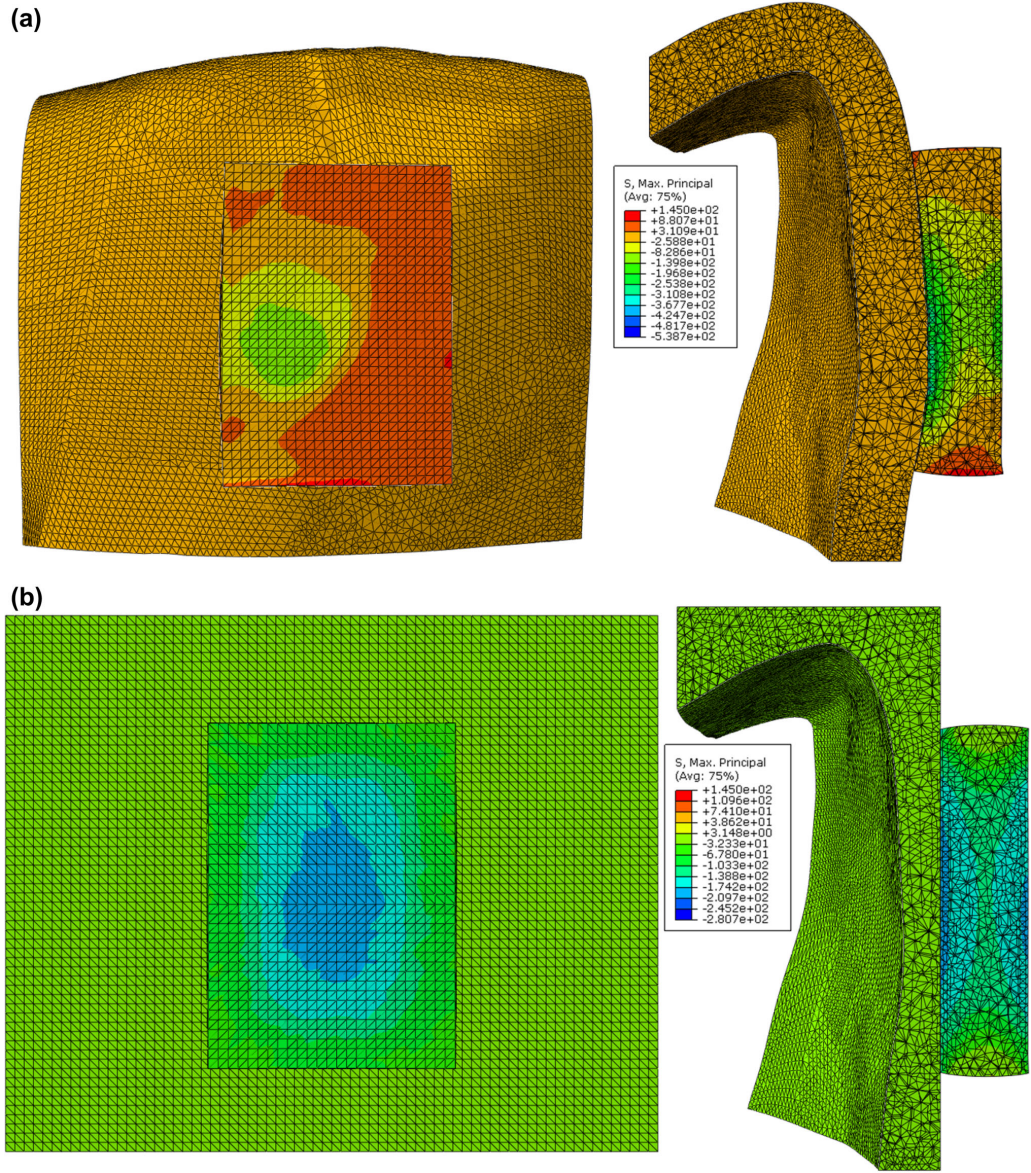
(a) Impact simulation of an LED mounted on an EVA shell with an overall thickness of 1.5 mm. Principal stress distribution of the LED in the deformed configuration at 0.57 ms. A maximum principal stress of 145 MPa was observed at the interface of the LED and EVA. (b) Impact simulation of an LED mounted on an EVA block with a minimum thickness of 1.5 mm. Principal stress distribution of the LED in the deformed configuration at 0.38 ms. A maximum principal stress of 119.5 MPa located at the lower right corner of the LED-EVA interface.

For the case with the EVA shell (Fig. 7a), the maximum principal stress caused by the impact was observed to be 145 MPa at 0.57 ms at the interface of the LED and EVA. For the case with the EVA block (Fig. 7b), the maximum principal stress caused by the impact was observed to be 119.5 MPa at 0.38 ms at the lower right corner of the LED-EVA interface. The stress concentration of the LED mounted on an EVA shell is more localised compared to the condition formed over a block. This is due to the fact that the area of contact provided by the shell is much smaller than that provided by the block EVA, hence giving rise to pressure points and yielding stresses significantly larger.

## Discussion

The results showed that the experimentally determined failure force (*F*_fail_) ranged from 117.2 to 272.2 N for those conditions that had components failure, with the thickness of mouthguard material and surface conformity having an effect on the protection of the electrical components.

### Failure Forces

The *F*_fail_ reported in this study provides a useful insight to how an electrical component would withstand low levels of impact. For an LED with no EVA protection located at a vertical offset of 3.5 mm (condition 2), this is considered the worst-case condition for the placement of an electrical component, since it does not have EVA for shock absorption as well as being subjected to the most extreme cantilevered load. The *F*_fail_ reported for this condition is 117.2 N. An analytical *F*_fail_ for a statically loaded LED was estimated to be 114.3 N, 2.5% lower than the experimentally obtained value. This was however not surprising, as the analytical value only accounted for a localised region reaching yield point, whilst the experimental value indicated a 50% chance of total fracture of the LED. For condition 2, the computationally simulated stress concentrations were orders of magnitude higher than the yield stress of epoxy resin (approximately 60 MPa). A linear elastic material law without damage model was used as the constitutive model for the LED, which did not yield under impact and therefore experienced stresses above typical yield stress. This simulation result is consistent with the experimental findings, where the LED can be expected to fail under the conditions in condition 2. The simulation result differed from the analytical calculations in terms of the location of the highest stress concentration, where the FE simulation of condition 2 showed a high stress concentration on the back of the LED rather than the front as predicted by the analytical calculations. This difference was due to the ways that the LED was loaded in both methods. In the analytical simulation, the load was simplified to only be distributed across the offset region, while in the FE simulations the load was distributed across the entire front of the face.

It was noted that for some of the experimental data points a “fail” occurred after repeated “no fails”. It is possible that the failure force could have been lowered for those specific cases due to material fatigue. However, the release angle of the pendulum (hence the impact load) was pseudo-randomised to converge towards the boundary between “fail” and “intact” states. The number of repeated impacts before a “fail” was also observed to be no more than four. Logistic regression was also used to estimate the failure force between the two states in order to further reduce bias. These approaches will have minimized the effect of repeated material fatigue.

The FE model is a useful tool for designing the placement of the electronic component within a smart mouthguard. However, two main limitations to the FE model are discussed below. Firstly, the boundary conditions used in the simulation may not be experimentally realistic. In the FE simulations the LED was modelled to be fully bonded to the dental models. However, during the experiments, in order to keep the contact surface of the dental model surface undamaged, the LED was fixed in position by securing its pins and not by means of adhesive. This can give rise to a significant amount of forces required to break the LED, as it was necessary for the impactor to push the LED towards the dental models before it made contact. The stresses computed by the FE (due to the fixed nodes) may be much higher than in the experimental condition. Furthermore, simplified boundary conditions were used here to study the effects of the EVA surface conformity. The material properties of the EVA were not fully taken into account, since the nodes on the front of the EVA material were all fixed in displacements. In reality EVA is likely to exhibit a viscoelastic response towards such impact loading. Here viscous effects important for understanding energy dissipation and history dependent responses were not modelled. Previous studies have shown that EVA with different amount of vinyl acetate exhibits different stress–strain relationship.^26^ Commercially available EVAs contain varying proportions of vinyl acetate and have been shown to absorb varying amounts of impact energy.^22^ This simplification is adequate for initial studies for surface conformity. When developing future FE models capable of predicting component failure, comprehensive material models are required that consider stress–strain curves at relevant strain rates of a specific EVA. Such development will require comprehensive experimental work on a material scale, such as pure tensile and compressive tests to validate the constitutive material model for EVA. Secondly, the simulated study used a linear elastic approximation for the LED, the material’s plastic and fracture behaviour were not captured. The FE simulations can only be used to observe regions that may be susceptible to damage. It is important to note that there is a wide range of material properties for epoxy and EVA reported to be dependent on composition and curing process in the literature.^6^,^36^ If a more accurate stress approximation is required, a more detailed material characterisation is necessary for the component of interest.

Both factors mentioned above could benefit from a test rig equipped with a high-speed imaging system that allows for capturing the true material contact and boundary conditions, as well as the material deformation. Techniques such as 3D digital image correlation show potential for the validation of subsequent computational models of the entire mouthguard.^21^ Nonetheless the presented simulations are very useful for the comparison of fracture patterns and the subsequent study on surface conformity.

### Mouthguard Thickness

The thickness of the mouthguard is key, since the shock absorption capability directly depends upon the thickness of the mouthguard material.^19^ According to the Academy for Sports Dentistry, “A Properly Fitted Mouthguard,” should “cover and protect both the teeth in the arch and the surrounding tissues” and have “a minimum of 3 mm thickness in the occlusal and labial areas”.^23^ Westerman *et al*. found that the optimal thickness for EVA mouthguard material is around 4 mm, where further increase in thickness improved shock absorption marginally, but this also gives a reduction in user comfort, increases speech restriction and interference with respiratory efficiency.^31^ The results from the protection study showed that the LEDs were adequately protected by two layers of 3 mm EVA sheets, up to a 6 mm pre-formed and on average 3.12 mm post-formed thicknesses (excluding thickness of electrical components). With the addition of embedded electronics, this could result in a mouthguard thickness between 5 and 6 mm post-formed. Although this thickness can ensure the LEDs’ functionality (all intact LEDs were electrically tested, and their functionality were verified) it can compromise wearer’s comfort. Further tests experimenting with one layer of EVA placed in front of or behind the LED were conducted and these results are included in the Supplementary Material, as this may be of interest when considering design optimisation of EVA thickness and component location.

It is important to note that this finding cannot be straightforwardly extrapolated for electrical components with a lower shock rating and a more complex electrical design, such as accelerometers and gyroscopes in terms of operational failure. Experimental reports on electronic packaging materials that are typical in accelerometers and gyroscopes shows the Young’s modulus to be higher than the potting resin used in this study.^15^ Therefore, in terms of the mechanical integrity of the packaging, one can postulate that such components will have a higher failure force. Research surrounding such sensor components largely focuses on the signal sensitivity, as the delicate mechanical components within such sensors are very sensitive to vibrational noise.^8^,^16^,^33^,^34^ In terms of practical noise reduction of such sensors in a field-scenario, rigorous signal processing and artificial intelligence may be necessary.^33^,^34^ Protection of power systems is also a major concern in the development of smart mouthguards. EVA is an insulator and it is important to note that the EVA layers never punctured even if the LED component failed. Furthermore, recent innovation with lithium batteries with magnesium electrodes shows promise in offering safety and operational stability even when damaged,^37^ it is therefore possible to design safe circuits that can withstand high levels of impact in the oral cavity.

From the conformity study, it is worth mentioning that experimentally increasing the EVA thickness to 1.5 mm did not alter the outcome of the LEDs on model B. This could be due to two factors. First, the conformity of the surface of which the LED is mounted on and second the thinning of the EVA post-forming meant that it was not providing adequate protection for the LEDs. Each factor will now be discussed in further details.

Custom-made mouthguards are made either by vacuum-forming or pressure-forming. The vacuum-forming process is more commonly used in industry, due to the ease of manufacture and reduced cost. The results from the computational study on surface conformity suggested that electrical components are more prone to damage when placed on surface with surface conformity similar to that of real dental geometry. While a levelled layer of EVA over a dental geometry can reduce the occurrence of stress concentrations within the component. Based on the finding of this study, a pressure-forming manufacturing process could be favoured for future smart-mouthguards designs.

It is widely reported that EVA is accustomed to thinning during fabrication, particularly in the incisal and cusp regions, where impacts are most common. Two mechanisms contribute to this effect: thinning by heating the material (droop) and thinning by stretching the material over the cusps and incisal edges.^31^ Calipers were used in a further study to measure the post-forming labial thickness of each mouthguard and a statistically significant difference (*t* test, *p* < 0.001) in the degree of thinning was observed between mouthguards formed over electronic components (48%) and those formed in the absence of electronic components (33%). This additional stretching of the EVA sheet is likely to stem from the sheet catching on the LED’s sharp edge, as it is draped onto the dental cast and therefore providing little or no protection to the LED component as in for conditions 3 and 6.

### Impact Energy

Finally, it is important to note that the maximum impact energy that is generated by this test rig is approximately 9.3 J, where the typical impact energy of a hockey ball can be approximated to be 31.8 J (assuming a drive speed of 20/ms and a ball mass of 159 g). Therefore, the results presented in this study should only be used for considering lower speed impacts. Further tests can be completed on a test rig with a high impact energy to evaluate the level of protection that is necessary for future smart mouthguard designed for hockey. The impact energy used in this study will be on the extreme end for many of the contact-sports that use mouthguards.

The focus on wellbeing for sport participation is essential, as more and more people rely on sport activity to keep themselves physically healthy. Safe sport participation reduces the need for medical and social intervention throughout the lifespan of people. Hockey England strongly encourages the use of mouthguards in adult fixtures and mandates their use in youth fixtures. Studies show that since 2000, 84.5% of players regularly wear mouthguards, whereas only 31.4% wore mouthguards before 2000.^30^ In order to have a safe and functioning smart mouthguard suitable for sports it is important to ensure structural safety of individual components and the connections between them. This study has shown that components could be safely added to mouthguards in order to increase the utility of this widely used safety equipment. This study has shown that components could be safely added to mouthguards at strategic location, such as the front of the incisor. Furthermore, the detailed methodology used here can be adapted for evaluating other specific loading scenario e.g. fatigue caused by chewing.^34^ Further studies can evaluate specific designs in order to further increase the adoption of in-body wearables.

## Electronic supplementary material

Below is the link to the electronic supplementary material.

Supplementary material 1 (PDF 178 kb)
