# Supplementary material for: Mechanical Safety of Embedded Electronics for In-body Wearables: A Smart Mouthguard Study
Source: Ann Biomed Eng. 2019 Apr 25;47(8):1725–37. doi: 10.1007/s10439-019-02267-4 (PMC6647539; doi:10.1007/s10439-019-02267-4)
Supplement: Supplementary file 1 — Supplementary material 1 (PDF 178 kb) [file 10439_2019_2267_MOESM1_ESM.pdf]

Supplementary Material

Impactor

A specialised hockey sliced impactor of thickness 19mm (Fig. 3) was fabricated from a polymer core hockey ball of diameter 230mm ($\pm$5mm). This ensured that the impactor could accurately target the LED embedded in the mouthguard. The hockey slice impactor was attached onto the pendulum of the impact rig with an internal stainless steel screw. A typical field hockey ball typically consists of a polyurethane (PU) or polyvinyl chloride (PVC) non-flexible shell, often shaped around a cork-polymer core or left hollow [3]. Although, the applied impactor design changed the core composition of the hockey slice, compared to a hockey ball in practice, the hardness of the impactor surface is similar to that of a hockey ball. It was therefore considered suitable for testing the effects of impact on the embedded electrical components. A mass of 139g was mounted on the pendulum to maximise the range of impact energy available from the test rig.

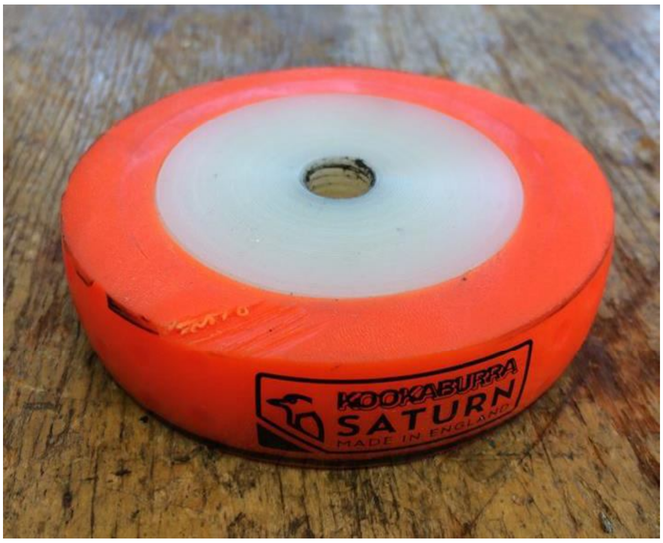

Figure S1 - Hockey ball slice impactor used in the pendulum test rig.

Impact acceleration

The experimental impact wave was approximated by half of a sine wave.

The inertia measurement unit (IMU) provides the release angle (θ_1) during static conditions based on the projection of the gravity vector on each of sensor axis. The IMU also provided the maximum angle reached post impact (θ_2). This information was then used to computed the impact velocity (v_1) and rebound velocity (v_2) by

$$v_i = \sqrt{2gL(1 - \cos\theta_i)} \qquad (1)$$

with *i* being 1 or 2, *g* representing 9.81 m/s^2 and a length of the pendulum (L) of .630 meters.

These velocities were taken forward to compute the maximum acceleration (a_{max}) as it was assumed that the impact wave shape resembles a half sine with a pulse width (T),

$$a_{max} = \frac{\pi}{T}\left(\frac{v_2 - v_2}{2}\right) \qquad (2)$$

It is expected that the equivalent mass impacting the LED is equal to the mass of the pendulum (1.81 kg). The maximum impact force (F_{max}) is therefore defined as:

$$F_{max} = ma_{max} = \frac{1.81 \cdot \pi\sqrt{2gL}\left(\sqrt{1 - cos\theta_2} - \sqrt{1 - cos\theta_1}\right)}{2T} \qquad (3)$$

with force given in Newton (N).

Theoretical failure force

An analytical solution for cantilever beam under uniformly distributed load was used to verify the magnitude of the experimental failure force for Condition 2.

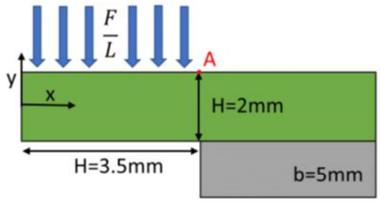

Figure S1 - LED modelled as a cantilever under a uniform load. Coordinate axis orientation originates at the tip of the cantilever.

Analysis of a beam under simple bending is defined as follows,

$$M(x) = \frac{\left(\frac{F}{L}\right) \cdot x^2}{2} \qquad (S.1)$$

where M is the bending moment, F is the impact force and L is the length of the beam. The maximum bending moment (M_{max}) is found at the base of cantilever($x = L$) can be expressed as the following:

$$M_{max} = M(x = L) = \frac{\left(\frac{F}{L}\right) \cdot L^2}{2} \qquad (S.2)$$

where M is Bending moment (Nm), F is Impact force (N) and L is Length of overhang (m). The bending stress (σ_{max}), calculated by Eq. ($S.3$), is expected to occur at the same location as M_{max} on the upper surface, expressed by Eq. ($S.4$), of the LED marked by 'A' in Fig. S1.

$$\sigma_{max} = \frac{M_{max} \cdot y_{max}}{I} \qquad (S.3)$$

$$y_{max} = \frac{H}{2} \qquad (S.4)$$

Here, I is the beam's second moment of area and H is the beam width.

Substituting Eq. (S.2), (S.3) and (S.4) gives:

$$\sigma_{max} = \frac{\frac{FL}{2} \cdot \frac{H}{2}}{\frac{bH^2}{12}} = \frac{3FL}{bH^2} \qquad (S.5)$$

where b is the beam depth. The tensile strength of epoxy resin is 60MPa. A theoretical F_{fail} can therefore be determined using Eq. $(A.5)$.

$$F_{fail} = \frac{bH^2\sigma_{max}}{3L} = \frac{0.005m \cdot (0.002m)^2 \cdot (60 \times 10^6 Pa)}{3(0.0035m)} = 114.3N \qquad (S.6)$$

Comparison of $F_{fail\,(Theory)}$ $(Theory)$ and $F_{fail\,(Exp)}$ for Condition 2 reveals an absolute error of 2.9N, a percentage error of 2.5%.

Mouthguard thickness

The thickness of the mouthguard is key to its effectiveness in trauma prevention, since the shock absorption capability directly depends upon the thickness of the material [1]. According to the Academy for Sports Dentistry, "A Properly Fitted Mouthguard," should "cover and protect both the teeth in the arch and the surrounding tissues" and have "a minimum of 3 mm thickness in the occlusal and labial areas" [2]. Westermann's found that the optimal thickness for EVA mouthguard material is around 4mm. Increasing the thickness further only improved shock absorption marginally, whilst user comfort, speech restriction and interference with respiratory efficiency was negatively influenced [4]. Several companies recommend the use of a 4mm mouthguard consisting of at least 2 layers of EVA for ball sports, such as hockey. In this study, a selection of mouthguard thicknesses ranging from 1.5mm up to 6mm (pre-formed) were tested. Mouthguards below the recommended thickness (3mm) were tested to guarantee that failures would be observed, permitting a threshold at which electronic component damage would occur.

Table S1: Summary of results for LED with one layer of EVA embedding arrangement parameters for the seven conditions.

**Condition**	**1**	**2**	**3**	**4**
	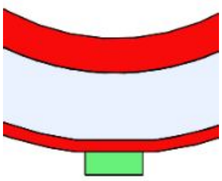	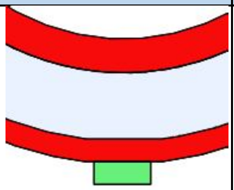	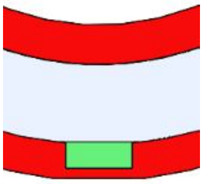	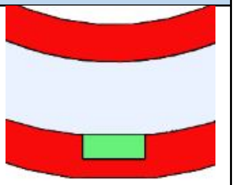
**Dental model**	A	A	A	A
**EVA thickness**	1.5mm	3mm	1.5mm	3mm
**Location**	Behind LED	Behind LED	In front of LED	In front of LED
**LED position**	3.5mm offset	3.5mm offset	3.5mm offset	3.5mm offset
F_{fail} (N)	210.0	295.8	131.5	141.3
β_0	-21.451	-13.278	-11.405	-53.049
β_1	0.073	0.101	0.081	0.195

Supplementary Material References

1. Maeda, Y., D. Kumamoto, K. Yagi, and K. Ikebe. Effectiveness and fabrication of mouthguards. *Dent. Traumatol.* 25:556–564, 2009.

2. Padavona, D. White House Concussion Summit Constituent Advocacy: Georgia AGD Healthy People 2020 Report Update AGD Members' Advocacy Efforts The Newsmagazine for the General Dentist. 42:, 2014.

3. Ranga, D., J. Cornish, and M. Strangwood. The role of materials and construction on hockey ball performance. *Eng. Sport 7* 1:435–442, 2009.

4. Westerman, B., P. M. Stringfellow, and J. A. Eccleston. EVA mouthguards: How thick should they be? *Dent. Traumatol.* 18:, 2002.
